# The Expression and Bioinformatics Analysis of Circular RNAs in Endometritis Mouse Uterus Tissues

**DOI:** 10.3390/molecules27123682

**Published:** 2022-06-08

**Authors:** Zhiqiang Li, Liying Shi, Qianqing Li, Jing Zhao, Wenfa Lu, Jun Wang

**Affiliations:** 1 Joint Laboratory of the Modern Agricultural Technology International Cooperation, Ministry of Education, Jilin Agricultural University, Changchun 130118, China; lizhiqiangsky@126.com (Z.L.); 18747551718@163.com (L.S.); lqq2020sunshine@163.com (Q.L.); 2 Key Lab of the Animal Production, Product Quality and Security, Ministry of Education, Jilin Agricultural University, Changchun 130118, China; 3 College of Animal Science and Technology, Jilin Agricultural University, Changchun 130118, China

**Keywords:** circular RNAs, endometritis, *Escherichia coli*, mouse

## Abstract

Previous studies have shown that circular RNAs are directly or indirectly involved in the occurrence of various diseases by regulating gene expression. However, the acting mechanism of circular RNAs in endometritis remains unclear. In this study, we successfully established an endometritis model in mouse using *Escherichia coli*; endometrial integrity was destroyed, inflammatory cells infiltrated and the expression of IL-6, IL-1β, TNF-α was significantly up-regulated. We analyzed and screened the circular RNA expression profiles between healthy and endometritis-stricken mice by the Illumina HiSeq platform, and used qRT-PCR method to verify the different expressions of circular RNAs. Gene ontology (GO) analysis showed that circular RNAs were mainly involved in biological processes such as the positive regulation of transcription from RNA polymerase POL II promoter and the negative regulation of cell proliferation. Kyoto Encyclopedia of Genes and Genomes (KEGG) analysis of circular RNAs target genes may be involved in the TGF-β signaling pathway. We verified the expression of TGF-β and its related factors; the mRNA of TGF-β1 and smad7 were significantly up-regulated in endometritis mouse (*p* < 0.01) and the protein expression level of p-smad3 was significantly decreased (*p* < 0.01). Finally, we constructed a circular RNAs–miRNA network to elucidate the potential regulatory relationship between two small molecules. This research may provide new ideas for circular RNAs in the treatment of endometritis.

## 1. Introduction

Endometritis is an inflammatory reproductive disorder caused by pathogenic microorganisms invading the uterus through the reproductive tract, blood or body fluid circulation, and is a common disease in the dairy farming industry [1]. At present, antibiotics are the main treatment for cow endometritis, but along with time, bacterial resistance and drug residues have attracted people’s attention [2]. Therefore, it is important to develop new therapeutic options to replace antibiotic therapy, and exploring the underlying pathogenesis of endometritis is the first step in identifying diagnostic and potential therapeutic targets.

Exonic circular RNAs (circular RNAs) were first discovered in pathogens and form covalently closed RNAs by back-splicing [3]. Circular RNAs have the characteristics of high stability and strong specificity [4]. In addition, they can also regulate gene expression and protein activity by adsorbing miRNA, thereby affecting protein transcription and synthesis [5,6]. They are a potential novel molecular marker and therapeutic target, which have attracted extensive attention of researchers. Previous studies have shown that circular RNAs are directly or indirectly involved in the occurrence of various diseases, such as rectal cancer [7], bladder cancer [8], hepatocellular carcinoma [9] and esophageal cancer [10]. In addition to cancer, circular RNAs are also involved in the occurrence of Alzheimer’s disease [11], myocardial fibrosis [12], atherosclerosis [13], osteoarthritis [14] and neuroinflammation [15]. Recent studies have found that circular RNAs can be involved in uterine diseases: Xu et al. [16]. found that there were 1258 up-regulated and 1061 down-regulated circular RNAs between normal and endometriosis patients, which acted as endometriosis activators and were potential therapeutic targets for the disease. In addition, circular RNA circFADS2 can interact with miR-643 to inhibit LPS-induced apoptosis of human endometrial epithelial cells and inhibit the occurrence of inflammation, proving that it plays an important role in endometritis [17]. Circular RNAs play important biological roles in different diseases, but their specific mechanisms in endometritis remain unclear.

In this study, we used *Escherichia coli* to establish a mouse endometritis model, compared and analyzed the uterine tissues of healthy and endometritis mice by Illumina HiSeq technology and annotated circular RNAs using bioinformatics methods. We screened the significantly differentially expressed circular RNAs and annotated their parental genes to explore their potential as molecular markers for the diagnosis of endometritis, and provide a theoretical basis for the underlying pathogenesis of endometritis.

## 2. Results

### 2.1. Establishment of a Mouse Model of Endometritis

**Figure 1:**
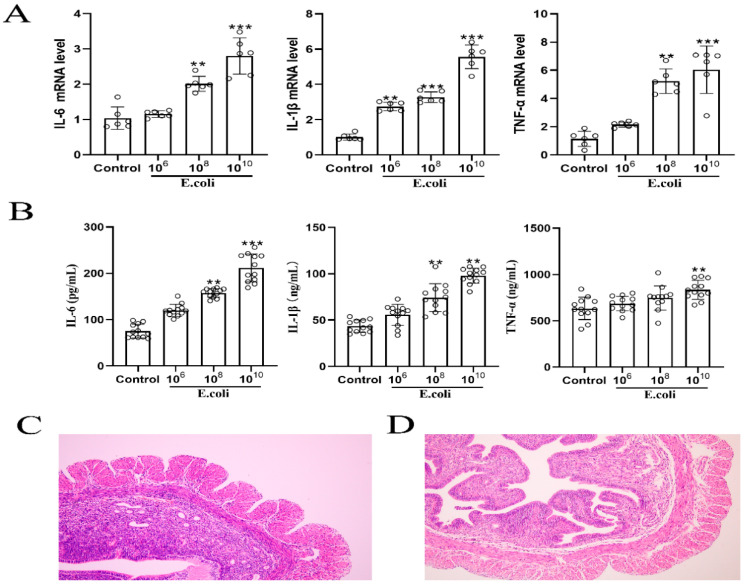
Infusion of *Escherichia coli* to establish a mouse model of endometritis. (**A**,**B**), Effects of different concentrations of *Escherichia coli* on mRNA and protein expression of IL-6, IL-1β and TNFα. **, *p* < 0.01, ***, *p* < 0.001, indicates a significant difference compared with the control group. Section of uterine tissue from healthy (**C**) and endometritis (**D**) mice.

Mice were injected with different concentrations of *Escherichia coli* (*E. coli*) for 24 h to establish a model of endometritis, and we detected the mRNA and protein expressions of IL-6, IL-1β and TNF-α in the mouse uterine tissues. As shown in Figure 1A,B, the mRNA and protein expressions of IL-6, IL-1β, and TNF-α were significantly up-regulated by 1010 CFU/mL *E. coli* (*p* < 0.01). Thus, 10^10^ CFU/mL *E. coli* was the optimal concentration for establishing a mouse model of endometritis. Next, we examined the pathological changes in the uterine tissues. The results showed that compared with normal mice, mice with endometritis had disrupted endometrial integrity, reduced gland numbers, increased intercellular spacing, and had marked inflammatory cell infiltration (Figure 1C,D).

### 2.2. Effect of Escherichia coli on NF-κB Signaling Pathway

**Figure 2:**
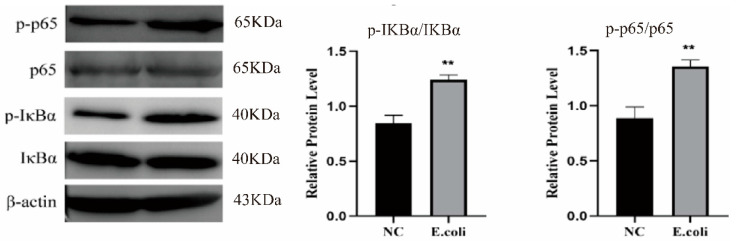
Effects of *Escherichia coli* on NF-κB. **, *p* < 0.01, indicating a significant difference compared with the NC group.

To further verify the successful establishment of the endometritis model, we also detected changes in the NF-κB signaling pathway. As shown in Figure 2, 1 × 10^10^ CFU/mL *E. coli* could significantly up-regulate the expression of p-p5, p-IκBα (*p* < 0.01). This showed that the model was successfully established and stable.

### 2.3. Identification and Type Statistics of Circular RNAs

**Figure 3:**
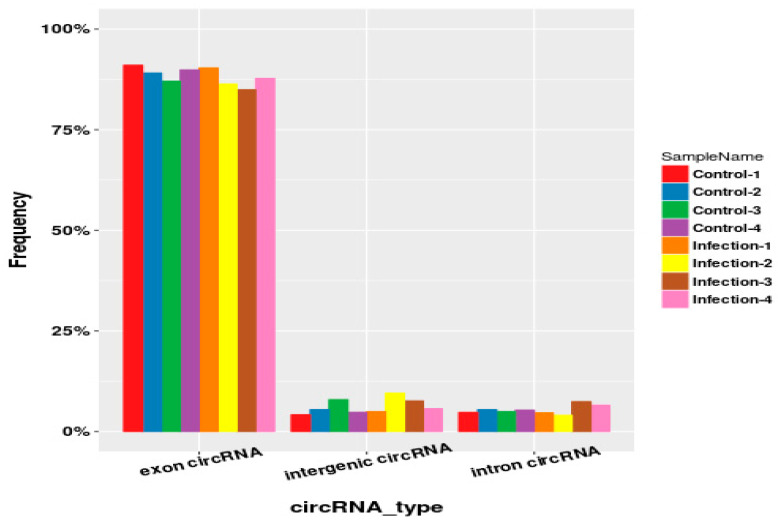
Main classifications of circular RNAs. Circular RNAs are mainly derived from exons, and the remaining sources are intergenic and introns.

As shown in Table 1, we found that each sample detected an average of 101,841,686.5 raw reads, of which more than 99% could be aligned to the mouse reference genome. We detected a total of 2356 circular RNAs, and 8% of the circular RNAs (190/2356) were included in the circBase database. Circular RNAs are mainly derived from exons, and the remaining sources are intergenic and introns (Figure 3).

### 2.4. Screening and Cluster Analysis of Circular RNAs

**Figure 4:**
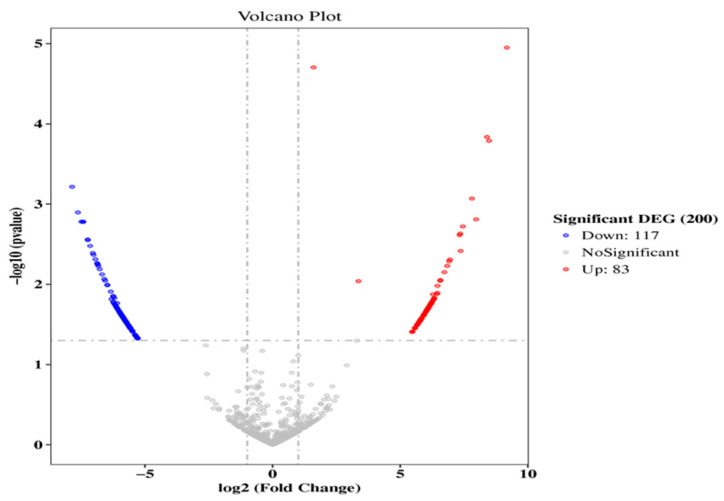
Differentially expressed circular RNAs in healthy and endometritis mouse. Blue means down-regulation, red means up-regulation, grey means not significant.

A total of 200 circular RNAs were differentially expressed in healthy and endometritis mice, including 83 up-regulated and 117 down-regulated. The uterine tissues of the two groups can be clearly distinguished by the volcano plot and hierarchical cluster analysis (Figure 4). The results showed that the data were reliable.

### 2.5. Validation of Selected Differentially Expressed Circular RNAs

**Figure 5:**
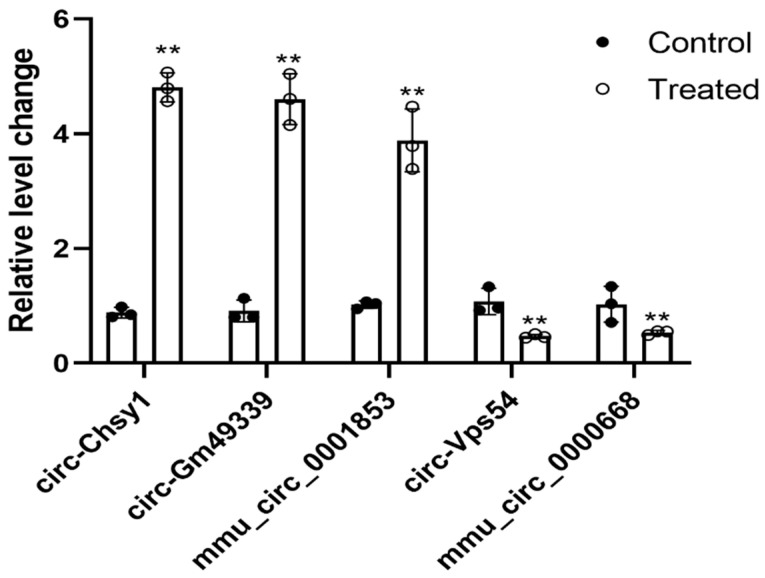
qRT-PCR to verify the expression of five circular RNAs. **, *p* < 0.01, indicating a significant difference compared with the control group.

In order to verify the differentially expressed circular RNAs, five of them were selected for verification based on the principles of high abundance and large differential fold (Table 2). As shown in Figure 5, compared with the control group, circ-Chsy1, circ-Gm49339, mmu_circ_0001853 were significantly overexpressed (*p* < 0.01), and circ-Vps54 and mmu_circ_0000668 were significantly underexpressed in the endometritis group (*p* < 0.01). The high-throughput sequencing results were consistent with the qRT-PCR verification results, confirming the reliability of the sequencing results.

### 2.6. GO and KEGG Analysis for the Circular RNAs

**Figure 6:**
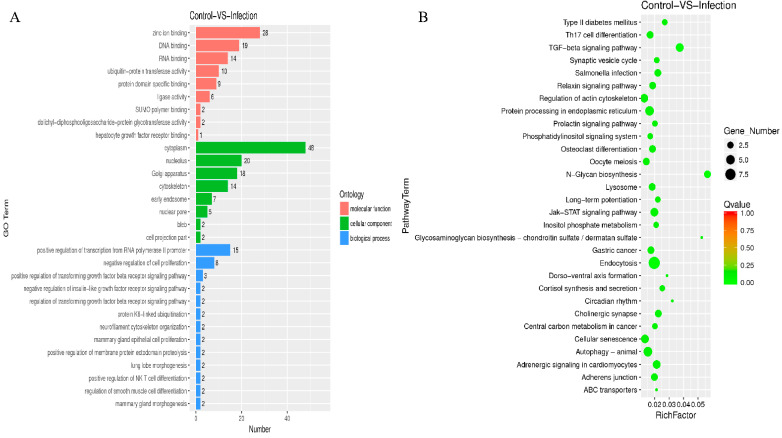
GO and KEGG pathway analysis of circular RNAs in healthy and endometritis mice. (**A**) GO analysis of the main biological roles of circular RNAs. (**B**) KEGG analysis of signaling pathways involved in circular RNAs.

To understand the role of circular RNAs in the pathogenesis of endometritis, we performed GO and KEGG analyses. The results showed that these circular RNAs were mainly involved in biological processes such as the positive regulation of transcription from RNA polymerase POL II promoter and the negative regulation of cell proliferation. In addition, they also played an important role in cellular components and molecular functions (Figure 6A). KEGG analysis results indicated that circular RNAs target genes may be involved in TGF-β, endocytosis, and N-glycan biosynthesis signaling pathways (Figure 6B).

### 2.7. The Role of TGF-β Signaling Pathway in Endometritis

**Figure 7:**
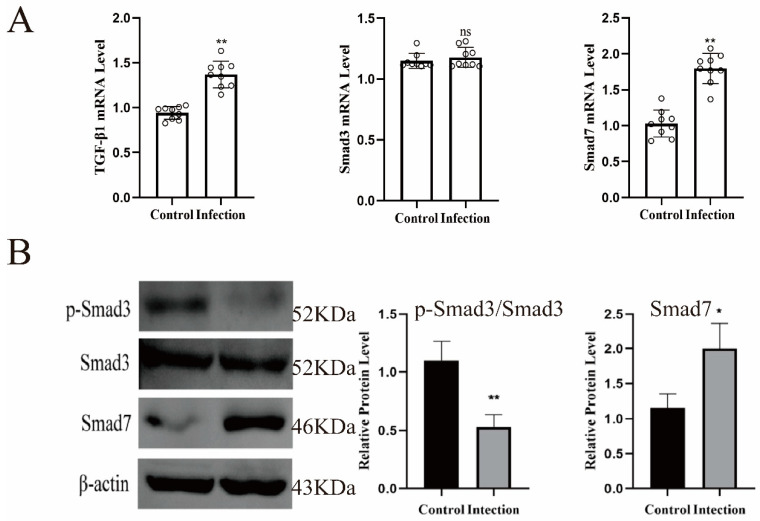
Effects of *Escherichia coli* on TGFβ signaling pathway. (**A**) Effect of *Escherichia coli* on the mRNA expression of TGFβ, smad3, smad7. (**B**) Effects of *Escherichia coli* on the protein expression of smad3 and smad7. *, *p* < 0.05, **, *p* < 0.01, indicating a significant or extremely significant difference compared with the control group.

Previous studies have shown that TGF-β1 could activate the phosphorylation level of Smad2/3 to induce the production of IκBα and negatively regulate the activation of NF-κB, thereby preventing the occurrence of inflammation [18]. Therefore, we verified the expression of TGF-β and its related factors. As shown in Figure 7A, the mRNAs of TGF-β1 and smad7 were significantly up-regulated in endometritis mice (*p* < 0.01), and smad3 also had a tendency to increase, but not significantly. However, the protein expression level of p-smad3 was significantly decreased and smad7 was significantly increased (Figure 7B, *p* < 0.01).

### 2.8. Circular RNAs–miRNAs Network

**Figure 8:**
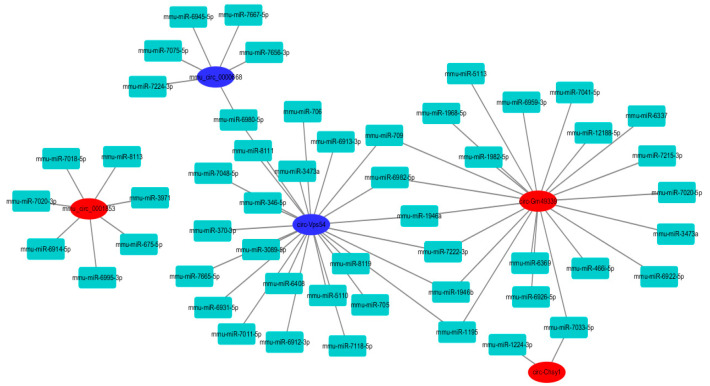
Construction of circular RNAs–miRNA network. Blue indicates down-regulated circular RNAs, red indicates up-regulated circular RNAs, linked to them are their targeted miRNAs.

Circular RNAs mainly reduce the effect of miRNAs on mRNA by adsorbing miRNAs and are involved in various diseases [16]. We predicted the miRNAs targeted by circular RNAs using the Miranda software, and constructed a circular RNAs–miRNA network to elucidate the close correlation and potential regulatory relationship between the two small molecules. As shown in Figure 8, the number of miRNAs targeted by circ-Vps54 is as high as 24 and the number of miRNAs targeted by circ-Gm49339 is 21, which are the two most important among the differentially expressed circular RNAs. In addition, six miRNAs, including mmu-miR-709, mmu-miR-6982-5P, mmu-miR-1946a, mmu-miR-7222-3p, mmu-miR-1946b, mmu-miR-1195, can target circ -Vps54 and circ-Gm49339, proving that there may be complex interactions between them, but further verification is needed.

## 3. Discussion

The initial defense of the endometrium against microbial infection and tissue damage depends on the innate immune system [19]. When infected, the integrity of the uterine tissue barrier will be disrupted, and the ability to resist inflammation continues to decline. The harm of endometritis is self-evident, and it is imperative to find new therapeutic targets. In this study, *Escherichia coli* was used to establish murine endometritis. First, the differentially expressed circular RNAs and their signaling pathways were screened by high-throughput sequencing detection, and preliminary verification was carried out. Finally, a circRNAs–miRNAs network was predicted and constructed to elucidate the close correlation and potential regulatory relationship of circRNAs–miRNAs.

With the in-depth study of various diseases, researchers have found that circular RNAs play an important role in a variety of diseases, and they are considered as potential novel molecular markers and therapeutic targets. Previous studies have shown that circRNA8073 can bind to miR-449a and inhibit its activity to increase the expression of Centrosomal protein55 (CEP55) and promote the proliferation of dairy goat endometrial epithelial cells through the PI3K/AKT/mTOR pathway. In addition, knockdown of Circ-8073 also induced apoptosis [20]. Zhang et al. [21] used microarray analysis to screen the circular RNA expression profiles of implantation sites and interimplantation sites in the endometrial tissue of early pregnancy mice; the author also screened differentially expressed circular RNAs and verified the important role of circular RNAs in embryo implantation. These studies demonstrated that circular RNAs play an important role in uterine tissue. In this study, we screened for differential circular RNAs, and through GO and KEGG analysis we found that these circular RNAs were mainly involved in biological processes such as the positive regulation of transcription from RNA polymerase POL II promoter and the negative regulation of cell proliferation. In addition, they also play an important role in cellular components and molecular functions. This is similar to previous findings; all these results demonstrated that circular RNAs played an important role in uterine disease.

In addition, we also found that circular RNAs exerted biological effects through the TGF-β signaling pathway. TGF-β works by inhibiting the production of cytokines by macrophages. It induces the production of IκBα by activating the phosphorylation of Smad2/3, and negatively regulates NF-κB to prevent inflammation [22]. Smad7 can bind to TGF-β1 type I receptors, compete with Smad2/3 for the catalytic site of phosphorylation, prevent the phosphorylation of Smad2/3, and significantly increase the production of inflammatory cytokines [23]. In this study, the mRNAs of TGF-β1 and smad7 were significantly up-regulated in endometritis mice, the protein expression level of p-smad3 was significantly decreased and smad7 was significantly increased. Previous studies found that TGF-β1 could inhibit the occurrence of inflammation by regulating the expression of microRNAs by activating smad3 [24]. TGF-β secreted by mesenchymal stem cells polarizes LPS-induced macrophages through the AKT/FOXO1 pathway and reduces the inflammatory response [25]. smad3 also promotes the anti-inflammatory protection of the heart by mediating a phagocytic phenotype [26]. Other studies have shown that TGF-β1/Smad3 signaling pathway and miR-21 also play important roles in renal fibrosis and inflammation [27]. These studies have found that TGF-β and smad3 play important roles in inflammation, which supported our findings.

In conclusion, our findings demonstrate that circular RNAs may mediate inflammatory responses through the TGF-β/smad3 pathway, which we consider to be potential targets for the treatment of endometritis. Due to its specific mechanism being still unclear, further research is needed.

## 4. Materials and Methods

### 4.1. Animal Treatment

Kunming White Mice (female, 8–10 weeks of age, weighing approximately 30–35 g) were purchased from Changsheng Biotechnology Company (Benxi, LiaoNing, China). *Escherichia coli* was provided by the Joint Laboratory of International Cooperation of the Ministry of Education of Modern Agricultural Technology (Changchun, Jilin, China). Briefly, all mice were housed in the same environment and randomly divided into two groups with 10 mice each after one week of acclimation. All mice were in estrus at the same time. Pregnant Mare Serum Gonadotropin (PMSG, 10 IU/mice, Changchun, Jilin, China) was injected on the first day, and human chorionic gonadotropin (HCG, 5 IU/mice) was injected on the second day. On the third day, Zoletil50 (50 mg/kg, Changchun, Jilin, China) was injected into the thigh muscle of each mouse. Then, 1 min after anesthesia, an indwelling needle (outer diameter: 0.8 mm) was inserted from the mouse reproductive tract, slowly extended to the cervix, and rocked left and right into the uterus. The control group and the *E. coli* group were injected with 25 μL of normal saline and the same amount of *E. coli* solution (10^10^ CFU/mL), respectively. The mice were inverted for 5 min and placed in a cage for observation, waiting for recovery.

### 4.2. Hematoxylin and Eosin

Uterine tissue was fixed with 4% paraformaldehyde, then embedded in paraffin, and 5-μm-thick sections were stained with hematoxylin and eosin (H&E) and morphologically analyzed according to standard protocols.

### 4.3. qRT-PCR

Total RNA from uterine tissue was extracted by Trizol method, Takara kit was used for reverse transcription, and fluorescence quantitative detection of related gene expression was performed. The primer sequence is shown in Table 3. Data analysis was calculated using the 2^−ΔΔCt^ method.

### 4.4. Enzyme-Linked Immunosorbent Assay (ELISA)

The released inflammatory cytokines TNF-α, IL-1β and IL-6 were detected with ELISA kits (Elabscience Biotechnology Co. Ltd., Wuhan, China). The optical density of the samples at 450 nm were measured using a Thermomax microplate reader (bio-tekEL, Winooski, VT, USA) following the instructions provided by the manufacturer.

### 4.5. Western Blot

The total protein of uterine tissue was extracted using RIPA lysate, and the sample quality was determined using the BCA kit. After transferring to nitrocellulose membrane, the membrane was blocked for 2 h, the matching antibodies were applied (Table 4), and after washing, protein bands were identified. The protein bands were detected by a chemisope imaging system (CLiNX Science Instruments, Shanghai, China) and the value for the control group was set as 100%.

### 4.6. Circular RNA Sequencing and Identification

Total RNA of each sample was extracted using Trizol Reagent according to the manufacturer’s instructions, followed by 1% agarose gel electrophoresis for identification of RNA integrity and assessment of genomic DNA contamination for each sample. A total of 1μg of total RNA was used following library preparation. Next generation sequencing library preparations were constructed according to the manufacturer’s protocol. Validation was performed using a Qsep100 (Biooptic, Taiwan, China) and quantification was performed by a Qubit 3.0 fluorometer (Invitrogen, Carlsbad, CA, USA). Then, libraries with different indices were multiplexed and loaded on an Illumina HiSeq 2500 instrument according to manufacturer’s instructions (Illumina, San Diego, CA, USA).

We processed the data with Cutadapt [28] (version 1.9.1) to obtain high quality clean data. Firstly, reference genome sequences and gene model annotation files of relative species were downloaded from genome websites, such as UCSC, NCBI, ENSEMBL. Secondly, BWA [26] (V0.7.5a-r405) was used to index reference genome sequence. Finally, clean data were aligned to reference genome via software BWA. Based on the SAM file, use CIRI [29] (V2.06) to predict the circular RNAs. The SAM files were scanned twice, and sufficient information was collected to identify and characterize circular RNAs. The abundance of circular RNAs was determined by the number of junction reads identified with CIRI tools. TargetScan (version 7.0, March 2018 release; http://www.targetscan.org/, 27 April 2022) tools were used to predict the target miRNA of identified circular RNAs.

### 4.7. Statistical Analysis

All data were from at least 3 experiments (*n* = 3) and were presented as mean ± standard deviation. Differences between groups were compared using one-way ANOVA and Student’s t tests. *p* < 0.05 was considered to be statistically significant.

## Figures and Tables

**Table 1:** Circular RNAs raw data statistics.

Sample	Clran Reads	Mapped Reads	Mapped Percent (%)	circRNAs Count
Control-1	105,483,211	105,260,546	99.79	692
Control-2	103,908,448	103,776,493	99.87	660
Control-3	103,188,307	103,012,390	99.83	642
Control-4	103,201,392	103,053,994	99.86	730
Infection-1	98,259,493	98,061,134	99.80	664
Infection-2	101,642,157	101,435,481	99.80	492
Infection-3	101,230,476	101,053,010	99.82	591
Infection-4	97,820,008	97,690,406	99.87	702

**Table 2:** The sequencing results of differential circular RNAs.

circRNA ID	GeneName	log2 Fold Change	*p*-Value	Regulation
circ-Chsy1	Chsy1	8.400876	0.00015	Ups
circ-Gm49339	Gm49339	8.480887	0.00016	Ups
mmu_circ_0001853	Tgfbr2	7.324954	0.00244	Ups
circ-Vps54	Vps54	−7.012614	0.00429	Down
mmu_circ_0000668	Mapk1	−5.975774	0.02260	Down

**Table 3:** Primer information.

Genes	Primer Sequence (5′–3′)	Tm (°C)
IL-6	F: CCACTTCACAAGTCGGAGGCTTA R: CCAGTTTGGTAGCATCCATCATTTC	60
IL-1β	F: TCCAGGATGAGGACATGAGCAC R: GAACGTCACACACCAGCAGGTTA	60
TNF-α	F: TATGGCCCAGACCCTCACA R: GGAGTAGACAAGGTACAACCCATC	60
β-actin	F: CTTCCTGGGCATGGAATCCT R: TTGATCTTCATTGTGCTGGGTG	60
Mus-circ-Gm49339	F: TGGAGTCCTGGTGTCATTCC R: CCCAACTGTTCAGCTCTGCA	62
mmu_circ_0001853	F: ACTCTGGAACATGCCGCTTC R: ATTGTCGCTGGCCATGACAT	62
Mus-circ-Chsy1	F: AGATGTGTCCGGAGATTCGC R: CCGGGAATTGTCTTGGACCA	62
Mus-circ-Ghr	F: CAGTCACCAGCAGCACATTT R: TTGCCATCACCTCCTTTCCC	62
mmu_circ_0000668	F: GGCACCAACCATTGAGCAAA R: TCAAGCCGATCAACACGTCA	62
Tgfb1	F: GTGGAAATCAACGGGATCAG R: ACTTCCAACCCAGGTCCTTC	58
Smad3	F: CCAGCACACAATAACTTGGA R: AGACACACTGGAACAGCGGA	61
Smad7	F: CAAACCAACTGCAGGCTGTC R: CCCCCAGGGGCCAGATAATTC	58

**Table 4:** Antibody information for Western blot.

Antibodies	Source and Cat No.	Dilution
Phosopho-NFκB p65	CST (3033S)	1:1000
NFκB p65	CST (8242S)	1:1000
Phosopho-IκBα	CST (2859S)	1:1000
IκBα	CST (4812S)	1:1000
Smad3	CST (9523S)	1:1000
Phosopho-Smad3	CST (9520S)	1:1000
Smad7	SANTA CRUZ (sc-365846)	1:1000
ACTB	ABclonal (AC038)	1:10,000
Peroxidase-conjugated Affinipure Goat Anti-Rabbit IgG(H + L)	Proteintech SA00001-2	1:10,000
Peroxidase-conjugated Affinipure Goat Anti-Mouse IgG(H + L)	Proteintech SA00001-1	1:10,000

## Data Availability

All participants confirmed it.

## Sample Availability

Samples of the compounds are not available from the authors.
